# National Food Affordability: A County-Level Analysis

**DOI:** 10.5888/pcd15.180079

**Published:** 2018-09-20

**Authors:** Anne Cafer, Georgianna Mann, Sujith Ramachandran, Michelle Kaiser

**Affiliations:** 1 Department of Sociology and Anthropology and Center for Population Studies, University of Mississippi, University, Mississippi; 2 Department of Nutrition and Hospitality Management, University of Mississippi, University, Mississippi; 3 Department of Pharmacy Administration, University of Mississippi, University, Mississippi; 4 School of Social Work, The Ohio State University, Columbus, Ohio

## Abstract

The purpose of this study was to explore the sociodemographic factors that contribute to food affordability across space, with specific emphasis on rural and urban differences in the United States. A regression analysis was used to predict food affordability from several predictors in rural and urban areas, with a subanalysis of Appalachian and Delta counties. Rural households had significantly higher food expenditures to income ratios compared with urban counties; Appalachian and Delta counties had the highest on average food expenditure to income ratio. Affordable food buffers vulnerable families against food insecurity and subsequent chronic health issues, which are especially relevant in the Appalachian and Delta counties.

## Objective

The purpose of this study was to explore contributors to food affordability across the rural/urban divide. This analysis also looked at how the most vulnerable regions, the US Delta and Appalachian counties, compare with the rest of the United States. Understanding contributors to food unaffordability across rural and urban counties, as well as in areas where poverty is persistent, is critical to health programming efficacy and to prevention of the development of chronic diet-related conditions among vulnerable populations. Specifically, the study examines the relationship between poverty, housing costs, access to food, and food affordability.

## Methods

Data from the American Community Survey (ACS), US Census Bureau, US Department of Agriculture’s Economic Research Service (USDA-ERS), and Feeding America’s 2016 *Map the Meal Gap* were used for this analysis. The justification and calculation of the food affordability measure, a percentage of median household income spent on food, are detailed in Cafer and Kaiser (1). The average Supplemental Nutrition Assistance Program (SNAP) benefits per participant were calculated for each county by using data from the Regional Economic Accounts Directorate of the Bureau of Economic Analysis. The percentage of each county’s residents paying more than 30% of their income on housing was obtained from the ACS 5-year estimates from 2011 through 2015. Total food stores per 1,000 residents and access to food stores, operationalized as the percentage of population living more than 1 mile from a food store in an urban area and more than 10 miles from a food store in a rural area, were both collected from the Food Environment Atlas. USDA designations for rural (1,257) and urban (1,885) counties were used. High-need counties were defined as those in the Appalachian (420 counties) or Delta (252 counties) regions that were identified as high-need counties by the Delta Regional Authority and the Appalachian Regional Commission (n = 560).

All data were aggregated using SAS version 9.4 (SAS Institute, Inc), and the analysis was performed for all 3,142 US counties. A linear regression model was run with PROC GLM, using food affordability as the dependent variable. The primary independent variable was the nature of the county. Several other predictors were included in the model, such as median household income, average SNAP benefits, and percentage of county’s residents below poverty guidelines. To further explore the rural/urban divide, we tested the nature of the county variable in an interaction with all the other predictors in the model. We dropped nonsignificant interactions from the model for reasons of parsimony.

## Results

Table 1 shows the descriptive statistics for food affordability and other county characteristics across rural and urban counties. Food affordability differed significantly (*P* < .001) for rural and urban counties in the United States, as shown in a *t* test (Table 1); people in rural counties pay nearly 19 percent of their household income on food compared with roughly 17 percent of income spent by household in urban counties. In other *t* tests, we found that households in rural counties, on average, had lower incomes, lower average SNAP benefits per participant, lower access to food stores, and higher percentages of individuals living below poverty guidelines. Urban households tended to have more unaffordable housing and fewer grocery stores per capita.

**Table 1:** Descriptive Characteristics of All Counties in the United States

Characteristic	Overall	Rural Counties	Urban Counties
Food affordability^a^^,^ b, mean (SD)	18.05 (4.97)	18.84 (5.33)	16.86 (4.08)
Annual household income, $^b^, mean (SD)	46,543.76 (12,081.96)	43,411.67 (9,594.84)	51,240.65 (13,789.80)
Percentage of households paying more than 30% of income on housing^b^, mean (SD)	27.7 (6.7)	25.8 (5.9)	30.6 (6.9)
Monthly SNAP benefits per participant, $^b^, mean (SD)	153 (16)	149 (17)	158 (13)
Total no. of food stores per 1,000 residents^b^, mean (SD)	0.26 (0.22)	0.30 (0.26)	0.19 (0.10)
Access to food stores, no. (%)^b^^,^ c	23.56 (20.25)	23.20 (24.68)	24.11 (10.55)
Percentage of individuals living below poverty guidelines^b^, mean (SD)	16.27 (6.47)	16.98 (6.71)	15.22 (5.94)
No. of high-need counties (food affordability in high-need counties^d^)	560 (17.82)	148 (11.77)	412 (21.86)

Abbreviations: SD, standard deviation; SNAP, Supplemental Nutrition Assistance Program.

a Percentage of median household income spent on food (1).

b *P* < .001, *t* test.

c Operationalized as the percentage of population living more than 1 mile from food store in an urban area and more than 10 miles from food store in a rural area.

d Counties in the Appalachian (420) or Delta (252) regions that were identified as high-need counties by the Delta Regional Authority and Appalachian Regional Commission.

The results of the linear regression (Table 2) revealed that after controlling for other covariates, food affordability is significantly predicted by nature of the county, median household income, affordability of housing, and poverty. The effect of poverty on food affordability was dependent on the nature of the county (*P* < .001). This interaction shows that an increase in the prevalence of poverty led to lower food affordability after controlling for other covariates, but this effect was much stronger in rural counties than in urban counties.

**Table 2:** Prediction of Food Affordability in the United States

Characteristic	Linear Regression Estimate (Standard Error)	*P* Value
Nature of county^a^		
Rural	0.0161 (0.0044)	<.001
Urban	1 [Reference]	
Median annual household income, in thousands of dollars	−0.0113 (0.0009)	<.001
Percentage of households paying more than 30% of income on housing	0.0730 (0.0130)	<.001
Average monthly Supplemental Nutrition Assistance Program benefits per participant	0.0171 (0.0056)	.003
Total no. of food stores per 1,000 residents	0.00657 (0.00425)	.88
Access to food stores^b^	0.00723 (0.0039)	.06
Percentage of individuals living below poverty guidelines	0.00455 (0.00018)	<.001
County designation		
Appalachian or Delta counties^c^	0.00128 (0.002)	.55
Other counties	1 [Reference]	
Nature of the county × percentage of individuals below poverty guidelines	−0.0014 (0.0002)	<.001

a As designated by the US Department of Agriculture.

b Operationalized as the percentage of population living more than 1 mile from food store in an urban area and more than 10 miles from food store in a rural area.

c Counties in the Appalachian (420) or Delta (252) regions that were identified as high-need counties by the Delta Regional Authority and Appalachian Regional Commission.

## Discussion

High food costs relative to other household budget items culminate in pervasive food insecurity, malnutrition, and obesity in low-income households. Affordable food buffers vulnerable families against these problems (2,3). This is particularly true for regions that have higher concentrations of low-income populations, such as the US Delta and Appalachian regions. This study revealed these regions to be particularly vulnerable to higher food expenditures, in part because these regions tend to lack the resources to address many issues of high food prices, low food access, or affordable housing (4). This vulnerability is compounded by the higher rates of obesity and related comorbidities in rural areas, specifically the Delta and Appalachian regions of the United States (5). The long-term implications are clear for these counties that often lack the types of preventive or clinical care necessary to manage these nutritionally driven diseases (6). Further programming should focus on increasing access and affordability of nutritious foods to prevent chronic disease as well as address housing issues that often limit the income available to purchase healthy foods (7). Specifically, this analysis points toward a true need, particularly in rural counties, for full-service grocery stores that provide healthier, more affordable food options compared with convenience and dollar stores. This study demonstrates this need to be a reality across the United States, not just for 1 region. Further studies should examine community coping mechanisms and perform in-depth primary analysis at the regional or state level. This analysis provides a national picture, but lacks the specificity of more localized analysis, which may account for different significant predictors than those found in previous studies.
